# Combined Effects of Vitamin D3 and Extracorporeal Shockwave Therapy on Osteoblast Differentiation via F-actin mediated Pdia3 Membrane Localization

**DOI:** 10.7150/ijms.127831

**Published:** 2026-07-22

**Authors:** Jai-Hong Cheng, Chi-Hsiang Hsu, Meng-Lun Tsai, Jeng-Wei Chen, Shun-Wun Jhan, Suhyun Lee, Chien-Yiu Huang, Shan-Ling Hsu

**Affiliations:** 1 Center for Shockwave Medicine and Tissue Engineering, Kaohsiung Chang Gung Memorial Hospital and Chang Gung University College of Medicine, Kaohsiung 833, Taiwan.; 2 Medical Research, Kaohsiung Chang Gung Memorial Hospital, Kaohsiung 833, Taiwan.; 3 Department of Orthopedic Surgery, Kaohsiung Chang Gung Memorial Hospital and Chang Gung University College of Medicine, Kaohsiung 833, Taiwan.; 4 Department of Pharmacy, College of Pharmacy, Woosuk University, Wanju 55338, Republic of Korea.

**Keywords:** vitamin D3, extracorporeal shockwave therapy, osteoblast, Pdia3-PKC signaling, F-actin

## Abstract

**Aim:**

1α,25(OH)₂D₃ (vitamin D3, VitD3) and extracorporeal shockwave therapy (ESWT) promote osteoblast differentiation and mineralization. Protein disulfide isomerase-associated 3 (Pdia3) mediates rapid membrane signaling of VitD3, but its interaction with ESWT remains unclear.

**Materials and Methods:**

MC3T3-E1 cells were treated with VitD3, ESWT, or both. Alkaline phosphatase activity and Alizarin red staining assessed osteoblast differentiation and mineralization. Pdia3 expression and signaling were evaluated by Western blotting and ELISA. Pdia3 distribution was analyzed by immunofluorescence and flow cytometry.

**Results:**

The actin polymerization inhibitor latrunculin B tested the role of F-actin. Combined VitD3 and ESWT significantly increased ALP activity, mineral deposition, Pdia3 expression, and plasma membrane localization versus single treatments. These effects were mediated by Pdia3-dependent protein kinase C (PKC) activation. Disruption of F-actin reduced Pdia3 membrane localization and abolished the combined osteogenic effects. ESWT promoted F-actin repolymerization, facilitating Pdia3 trafficking to the membrane.

**Conclusion:**

VitD3 and ESWT exert enhanced combined effects on osteoblast differentiation and mineralization by promoting F-actin mediated Pdia3 membrane localization and activating the PKC pathway. The integrity of the actin cytoskeleton is essential for these effects.

## Introduction

Protein disulfide isomerase family a member 3 (Pdia3), also named ERP57, belongs to the protein disulfide isomerase (PDI) family and is a protein disulfide isomerase enzyme with multifunctional roles 1. Pdia3 is expressed mainly in the endoplasmic reticulum (ER); however, this protein has also been found in other cellular compartments, such as the nucleus, cytoplasm, mitochondria, and cell membrane 2, 3. Pdia3 has been reported to function as an ER chaperone that binds nascent proteins and facilitates their proper folding 4. Additionally, it has been identified as a transcription factor that regulates the expression of interleukin enhancer-binding factor 3 (ILF3) and dyskerin pseudouridine synthase 1 (DKC1) 1, 5. In terms of mitochondrial functions, Pdia3 is involved in pro-apoptotic functions and modulates Ca^2+^ crosstalk between the ER and mitochondria 6, 7. In the role of bone development, Pdia3 is a membrane receptor for 1α,25-dihydroxyvitamin D3 (1α,25(OH)_2_D_3_), which is critical for calcium homeostasis and modulates the proliferation and differentiation of osteoblasts 8. Mutation of the Pdia3 gene results in musculoskeletal phenotype changes in the growth plate and long bone formation, suggesting its important role in bone formation 9.

Pdia3 appears to participate in controlling 1α,25(OH)_2_D_3_-induced phospholipase C (PLC), PKC and extracellular signal-regulated kinases 1 and 2 (ERK1/2) activation in addition to downstream responses to gene transcription for bone formation and development 10-12. 1α,25(OH)_2_D_3_ is an active form of VitD3 and is well known to affect calcium homeostasis in the development of growth plate and bone structure 13. Chronic vitamin D deficiency inhibits bone matrix synthesis and cartilage growth in the growing organism. Vitamin D affects the maturation of osteoblasts and growth plate chondrocytes through binding with the vitamin D receptor (VDR) and Pdia3 14. Pdia3 is a receptor for the rapid membrane signaling pathway that interacts with 1α,25(OH)_2_D_3_ and is involved in Wnt Family Member 5a (Wnt5a) signaling that regulates genes associated with cell proliferation and differentiation 1. Extracorporeal shockwave therapy (ESWT) has been reported to induce the expression of Pdia3 and modulated proteins involved in rapid membrane signaling pathways, such as extracellular signal-regulated protein kinase 1 (ERK1), osteopontin (OPN), and alkaline phosphatase (ALP), in a rat model of knee osteoarthritis (OA) 15. However, the precise molecular mechanisms by which ESWT regulates Pdia3 remain unclear.

Extracorporeal shock wave therapy (ESWT) is a well-established, noninvasive treatment for musculoskeletal disorders that exerts its biological effects through mechanotransduction 16. ESWT is first reported to improve bone healing in the treatment of nonunion fractures 17. The biological effects of ESWT on bone healing include the upregulation of angiogenic markers, such as vascular endothelial growth factor (VEGF), proliferating cell nuclear antigen (PCNA), and endothelial nitric oxide synthase (eNOS), as well as the osteogenic marker bone morphogenetic protein-2 (BMP-2) 18. Osteoblasts are activated by ESWT to promote the expression of integrins and the phosphorylation of focal adhesion kinase (FAK). Integrins and FAK are the significant factors in the mechanotransduction pathway, which physically stimulates biological reactions in cells 19. ESWT has chondroprotective effects and promotes bone remodeling in patients with knee OA 20. Many factors, including transforming growth factor beta-1 (TGF-β1), BMP2, insulin-like growth factor 1 (IGF-1), Pdia3, ERK1, osteopontin, ALP and Wnt5a, stimulate subchondral bone remodeling in knee OA animal model after ESWT 15, 21-23. In particular, Pdia3 is first reported to be enhanced by ESWT and is correlated with the treatment of knee OA 15.

Current studies in OA have highlighted the importance of mechanotherapy, which utilizes mechanical stimuli to modulate cellular behavior and tissue remodeling. Therefore, ESWT has emerged as a promising non-invasive modality capable of influencing both cartilage and subchondral bone regeneration through mechanotransduction pathways. Previous studies have demonstrated that ESWT can reduce pain and improve joint function of patients with knee OA. These therapeutic effects have been attributed to the modulation of inflammatory responses, the stimulation of chondrocyte activity, and the promotion of subchondral bone remodeling 24. Notably, recent studies further indicate a dose-response relationship, where higher energy levels of ESWT are better improvements in clinical outcomes with Western Ontario and McMaster Universities Arthritis Index (WOMAC) and Visual Analogue Scale (VAS), showing the therapeutic relevance of mechanical stimulation intensity 25.

Beyond its demonstrated clinical efficacy, recent high-impact studies have substantially advanced our understanding of the molecular and cellular mechanisms underlying ESWT as a mechanotherapeutic intervention. For instance, the radial ESWT can attenuate osteoblast senescence in subchondral bone, a key pathological feature in early OA, thereby suggesting a role in modifying disease progression rather than merely providing symptomatic 26. Complementary investigations further support that ESWT modulates the joint microenvironment by regulating cellular metabolism, promoting tissue regeneration, and influencing bone-cartilage crosstalk, reinforcing its classification as a mechanobiological therapy 27, 28. These findings indicate that ESWT not only improves clinical symptoms but also exerts disease-modifying effects through mechanotransduction-driven cellular responses, expanding its applicability in OA treatment. However, the relationships among VitD3, Pdia3 and ESWT in osteoblast maturation are still unknown. This study aimed to elucidate the role of Pdia3 in osteoblast differentiation and mineralization following VitD3, as well as its relationship with ESWT.

## Materials and Methods

### Cell culture

MC3T3-E1 subclone 4 (ATCC CRL-2593) cells were purchased and cultured in proliferation medium (alpha-MEM, Gibco, NY, USA) supplemented with 10 % fetal bovine serum (Gibco, NY, USA) and 100 units/mL penicillin/streptomycin (Gibco, NY, USA) in a humidified incubator at 37 °C with 5 % CO_2_ for the experiments.

### MC3T3-E1 cell differentiation and mineralization

Starting at 70 % confluence, the differentiated medium (DM) was supplemented with 100 μg/mL ascorbic acid (Merck, Darmstadt, Germany) and 10 mM β-glycerophosphate (Sigma-Aldrich, MO, USA) to support the differentiation of preosteoblasts into mature osteoblasts. The DM was changed every three days to maintain optimal cell conditions. The cells were cultured in DM for either 2 or 4 weeks to induce mineralization. Additional cell cultures were treated with 1α,25(OH)_2_D_3_ (Active vitamin D3; VitD3) at a concentration of 1x10^-12^ M (Sigma-Aldrich, St. Louis, MO) 29 and Latrunculin B (Abcam, Boston, MA, USA) at a concentration of 1 μM for the experiments 30.

### Alkaline phosphatase activity and alizarin red staining

The MC3T3-E1 cells from each group were cultured in 100 mm diameter culture dishes and trypsinized to a concentration of 10^6^ cells/mL in Eppendorf tubes. To prepare the lysates, 200 µL of PBS+Trion-X (1 %) was added, and the cells were vigorously pipetted after washing with cold PBS. The solution was further broken down by syringing through a 0.6 mm needle and then stored at -80 °C with 1X protease inhibitor cocktail (Sigma Aldrich, MO, USA). The ALP activity in the lysates was determined by measuring the absorbance of the reaction mixture containing p-nitrophenyl phosphate at 450 nm via spectrophotometry (Molecular Devices, CA, USA). All the samples were analyzed in triplicate.

The mineralized cells were fixed with 4 % paraformaldehyde at 25 °C for 20 minutes, washed with 5 mL of 1× PBS and stained with 1 mL of 0.5 % alizarin red staining solution at 25 °C for 15 minutes according to the manufacturer's instructions (Thermo Fisher Scientific, IL, USA). The cells were washed and shaken with distilled water for 6 minutes, after which the red mineralized nodules were visualized and counted via a CCD microscope (Carl Zeiss AG, Oberkochen, Germany). All the staining data were repeated three times.

### Alizarin red quantification

The mineralization of differentiated MC3T3-E1 cells was assessed via Alizarin red quantification with a commercial kit (TMS-008, Merck KGaA, Darmstadt, Germany). Cells grown in 24-well plates were treated with 400 μL of 10 % acetic acid per well for 30 minutes. After the cells were gently scraped and transferred to microcentrifuge tubes, the tubes were vigorously vortexed for 30 minutes. The tubes were then heated to 85 °C and centrifuged for 15 minutes. Quantitative analysis of Alizarin red was conducted by measuring the absorbance at 504 nm via a spectrophotometer from Molecular Devices. Each sample was tested three times in the experiment.

### Extracorporeal shockwave therapy

MC3T3-E1 cells, differentiated to a density of 8x10^6^ cells, were suspended in a 15-mL centrifuge tube containing 6 mL of medium for shockwaves (SWs) treatment. The tube was sealed with sterilized parafilm, and the cells in the medium were exposed to SWs via a DUOLITH SD1 device (Supplemental Figure 1A; Storz Medical AG, Tagerwilen, Switzerland) at focused energy flux densities ranging from 0 to 0.45 mJ/mm^2^, with 1000 impulses at 4 Hz for cell viability experiments (MTT assay, Supplemental Figure 1B) as modification from previous studies 19, 31. The optimized ESWT dosage of 0.25 mJ/mm^2^ with 1000 impulses at 4 Hz was subsequently used for the experiments (Supplemental Figure 1C).

### Experimental design for determining the Pdia3 distribution

The experimental design is illustrated in Figure 2A. The experimental groups were divided as follows: the growth medium (GM) group consisted of MC3T3-E1 cells cultured with GM. In the DM group, MC3T3-E1 cells were treated with DM for one week (step 1 - week 1) to induce cell differentiation. The cells were subsequently treated with DM for the second week (step 2 - week 2) until they were harvested at 6 hours (hr), 12 hr, 24 hr, 48 hr, 72 hr and 1 week. In the ESWT group, MC3T3-E1 cells were treated with DM for one week (step 1 - week 1) to induce cell differentiation. On the 8^th^ day, the cells subsequently received ESWT. The cells were then cultured in DM until they were harvested at 6 hr, 12 hr, 24 hr, 48 hr, 72 hr and 1 week. In the VitD3 group, MC3T3-E1 cells were treated with DM and vitamin D3 (1x10^-12^ M) for one week (step 1 - week 1) to induce cell differentiation as modification from previous report for using the concentration of VitD3 29. The cells were subsequently treated with VitD3 for the second week (step 2 - week 2) until they were harvested at 6 hr, 12 hr, 24 hr, 48 hr, 72 hr and 1 week. The VitD3+ESWT group involved treating MC3T3-E1 cells with DM and vitamin D3 (1x10^-12^ M) for one week (step 1 - week 1) to induce cell differentiation. On day 8, the cells subsequently received ESWT. The cells were then cultured in VitD3 for the second week (step 2 - week 2) before being harvested at 6 hr, 12 hr, 24 hr, 48 hr, 72 hr and 1 week.

The F-actin inhibition study were presented in Figure 6A. The cells were divided into four groups as described in Figure 6A and cultured for 2 hours. Subsequently, latrunculin B was added at a concentration of 1 μM for 3 hours as modification from previous study 30. The culture medium containing latrunculin B was then replaced with DM, and the cells were cultured for an additional hour.

### MTT assay for cell viability

MC3T3-E1 cells were cultured in GM for 24, 48, or 72 hours and subjected to cell viability analysis via the 3-(4,5-Dimethylthiazol-2-yl)-2,5-diphenyltetrazolium bromide (MTT) assay. The cells were seeded at a density of 10^4^ cells per well in 96-well plates for the cell growth assay. The MTT assay was performed as follows: 100 μL of MTT solution (1X) was added to each well, and the plate was then incubated at 37 °C for 4 hours. Subsequently, 100 μL of dimethyl sulfoxide was added to solubilize the formazan crystals. The absorbance intensity was measured at 495 nm with a reference wavelength of 620 nm via a spectrophotometer, and the relative cell viability was relative to that of the untreated control cells. Each sample was analyzed at least three times in the experiment.

### Trypan blue assay for cell mortality

MC3T3-E1 cell mortality was assessed via an automatic cell counter (Cellometer Auto T4, Nexcelom Bioscience, Lawrence, MA) following staining with Trypan blue. The cells were subsequently cultured in GM for 24, 48, or 72 hours before being trypsinized with EDTA-Trypsin (Thermo Fisher Sci, MA, USA). The trypsinized cells were then mixed with Trypan blue, and 20 μL of the mixture was loaded into chamber chips for quantification of dying cells.

### Immunofluorescence staining

The cells cultured on glass coverslips were fixed with 4 % paraformaldehyde for 5 minutes and then washed with PBS. For plasma membrane-specific Pdia3 staining, the cells were incubated in blocking buffer for only 1 hour (with no detergent treatment). Primary antibodies against Pdia3 at a dilution of 1:200 (ab13506, Abcam, MA, USA) were applied and incubated for 1 hour, followed by incubation with secondary antibodies with fluorescein isothiocyanate for 1 hour. For F-actin staining, the cells were treated with PBS containing 0.2 % Triton X-100 (Sigma-Aldrich, MO, USA) for 15 minutes at room temperature, followed by incubation in blocking buffer for 1 hour. Primary antibodies against F-actin at a dilution of 1:500 (ab130935; Abcam, MA, USA) were used for incubation for 1 hour, followed by incubation with secondary antibodies (rhodamine) for 1 hour. The glass coverslips were sealed with immunofluorescent mounting solution and examined under a fluorescence microscope equipped with a UV laser (ZEISS, AxioPlan 2; Carl Zeiss MicroImaging LLC, Germany) using different emission and excitation wavelengths. The percentage of positively expressing cells, termed Pdia3%-positive cells were calculated by ImageJ IHC profiler plugin (NIH, Bethesda, MD, USA).

### Plasma membrane protein-specific flow cytometry assay

The cells were trypsinized to obtain approximately 1x10^6^ cells and washed twice with fluorescence-activated cell sorting (FACS) buffer (PBS+5 % FBS), followed by resuspension in 100 μL of FACS buffer. Next, 2 μL of Pdia3 antibody (ab13506, Abcam, MA, USA) was added, and the cells were incubated for 60 minutes at room temperature. After being washed with FACS buffer, the cells were fixed with 4 % paraformaldehyde for 10 minutes, washed with cold PBS, and then incubated with a FITC-conjugated secondary antibody at a dilution of 1:200 (Alexa 488, Thermo-Fisher Sci., IL, USA) for 60 minutes. The mean fluorescent intensities (MFIs) were calculated via FlowJo software (BD Biosciences, CA, USA), and the relative MFI (MFI of the test sample/MFI of the isotype sample) were calculated for comparing Pdia3 levels 32. A Na^+^/K^+^-ATPase antibody (bsm-52485R, Biorbyt, Cambridge, UK) was used as a positive control at a ratio of 1:50, and an isotype mouse IgG1 ab (562026, BD Biosciences, CA, USA) was used as a negative control.

### Medium fluorescence intensity by plasma membrane protein-specific flow cytometry

A total of 5×10^4^ cells were collected at 6, 12, 24, 48 and 72 hours after ESWT. The cells were washed with flow buffer (0.05 % FBS and 10 % sodium azide in PBS) and labeled with an anti-Pdia3 monoclonal antibody (Sigma-Aldrich, MO, USA) for 1 hour of incubation. The cells were then incubated in the dark at 4°C for 1 hour with a secondary antibody. All MC3T3-E1 cells gated from the cellular debris via forward scatter/side scatter (FSC/SSC) were analyzed for the Pdia3 expression population first. The mean fluorescence intensity (MFI) of the cells was measured via laser excitation at the FITC wavelength (FL-1 channel) (BD Biosciences, CA, USA). The experiment was performed in triplicate, and 50,000 cells were assayed for each sample. The flow analysis and graphs were generated with FlowJo software (BD Biosciences, CA, USA).

### Cell membrane extraction

MC3T3-E1 cells were subjected to cell membrane extraction. Lysates from cells cultured in DM, ESWT, VitD3, and VitD3+ESWT groups were obtained and separated into cytosolic and plasma membrane fractions via the Plasma Membrane Protein Extraction Kit (ab65400, Abcam, MA, USA) following the manufacturer's instructions. Briefly, the cells were collected and lysed via a homogenizer in lysis buffer. The cell lysates were then centrifuged and filtered to obtain the pure membrane fraction. The extracted proteins were subjected to 7.5 % SDS-PAGE and analyzed by immunoblotting. Na^+^/K^+^-ATPase was used as a marker for the plasma membrane.

### Western blot

The lysates of the samples were quantified for their concentrations, separated by SDS-PAGE, and then transferred to polyvinylidene difluoride membranes (Bio-Rad Laboratories, CA, USA). The membranes were blocked with 5 % non-fat milk for 1 hour and incubated with primary antibodies targeting Pdia3 at 1:2000 (HPA003230, Sigma-Aldrich, MO, USA), BMP4 at 1:1500 (ab29973, Abcam, MA, USA), Runx2 at 1:1000 (ab23981, Abcam, MA, USA), OCN at 1:500 (PA5-96529, Invitrogen, CA, USA), ERK1/2 and Phospho-ERK1/2 at 1:1000 and 1:500 (Cell Signaling, MA, USA), β-actin at 1:1000 (Santa Cruz Biotech., TX, USA), and Na^+^/K^+^-ATPase at 1:1000 (bsm-52485R, Bioss Inc., MA, USA). Finally, the signal intensities were measured by ImageJ software (NIH, Bethesda, MD).

### Protein kinase C activity assay

The protein samples were prepared from cultured cells, isolated, and diluted to 1:30 in dilution buffer. PKC activity was analyzed via the PKC Kinase Activity Assay Kit (ab139437; Abcam, MA, USA) following the manufacturer's instructions. The protein concentration of the samples and the absorbance (OD at 450 nm) values for PKC activity were determined via a microplate reader (Molecular Devices) via the following equation: PKC activity = (ODsample - ODblank)/protein quantity.

### Statistical analysis

SPSS statistical software (Version 17.0, SPSS Inc., Chicago, IL, USA) was used for statistical analysis. The significant differences among the groups were compared via one-way ANOVA with Dunnett's post hoc test for parametric data, with P < 0.05, P < 0.01 and P < 0.001.

## Results

### ESWT and VitD3 have combined effects to enhance osteoblast differentiation and mineralization

**Figure 1:**
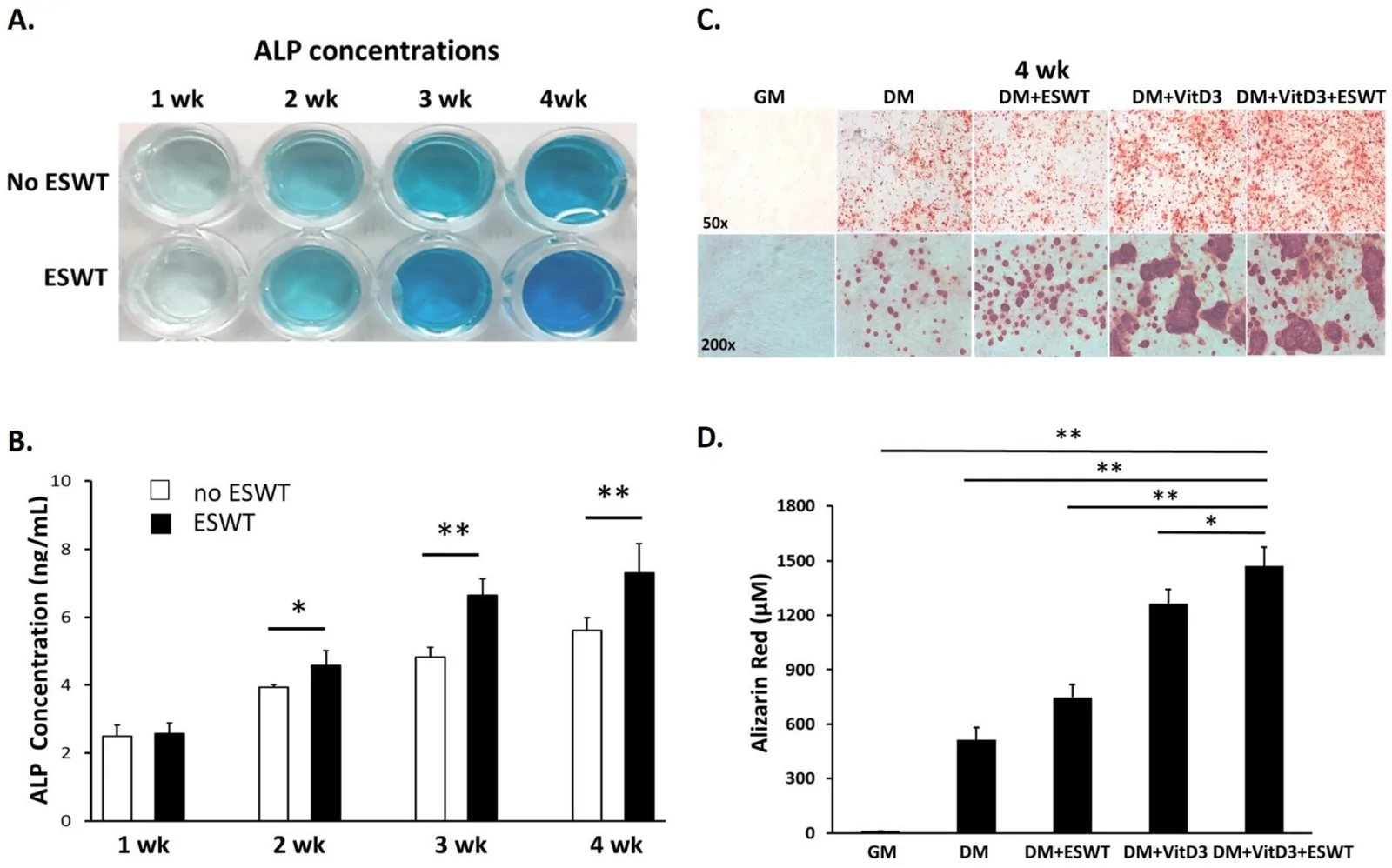
** Effects of ESWT on osteogenic differentiation and mineralization in MC3T3-E1 cells were analyzed via colorimetric and Alizarin red staining.** (A) Representative images showing ALP activity with varying intensities of coloration, indicating different ALP concentrations. (B) Quantitative analysis of ALP activity in the ESWT and non-ESWT groups over a time course of 1 to 4 weeks (wk). The data revealed greater ALP activity in the ESWT group than in the control group. (C) Microscopic visualization of calcium nodule formation in different experimental groups at 50× magnification (upper panel) and 200× magnification (lower panel) after 28 days of culture. (D) Quantification of Alizarin red staining, indicating calcium deposition. Compared with the other experimental groups, the DM+VitD3+ESWT group presented the highest concentration of calcium bicarbonate, with significant differences. DM refers to differentiated medium. The statistical analyses were significant at ^*^P < 0.05 and ^**^P < 0.01.

The optimal dosage of ESWT (0.25 mJ/mm^2^, 1000 impulses, 4 Hz) for osteoblast differentiation was determined, as shown in Supplemental Figure 1. Preosteoblastic MC3T3-E1 cells were induced with osteogenic medium, and the effects of ESWT and VitD3 on differentiation were assessed after the experiments (Figure 1). We found that ESWT group showed significantly greater ALP activity at 2 wk (P < 0.05), 3 wk (P < 0.01) and 4 wk (P < 0.01) than without ESWT (Figure 1A and 1B). In the alizarin red staining, DM+ESWT (P < 0.01) and DM+VitD3 (P < 0.01) groups revealed increasing calcium deposits in cells compared with the DM group at 4 wk (Figure 1C and 1D). As shown in Figure 1C, differentiated osteoblasts exhibited an elongated, filamentous morphology accompanied by extensive calcium deposition. Quantitative analysis demonstrated that the DM+VitD3+ESWT group showed significantly greater calcium deposition than the other experimental groups (P < 0.05 or P < 0.01), suggesting that the combined treatment with ESWT and VitD3 enhances osteoblast differentiation and mineralization.

### The expression and distribution of Pdia3 during osteoblast differentiation after treatment

**Figure 2:**
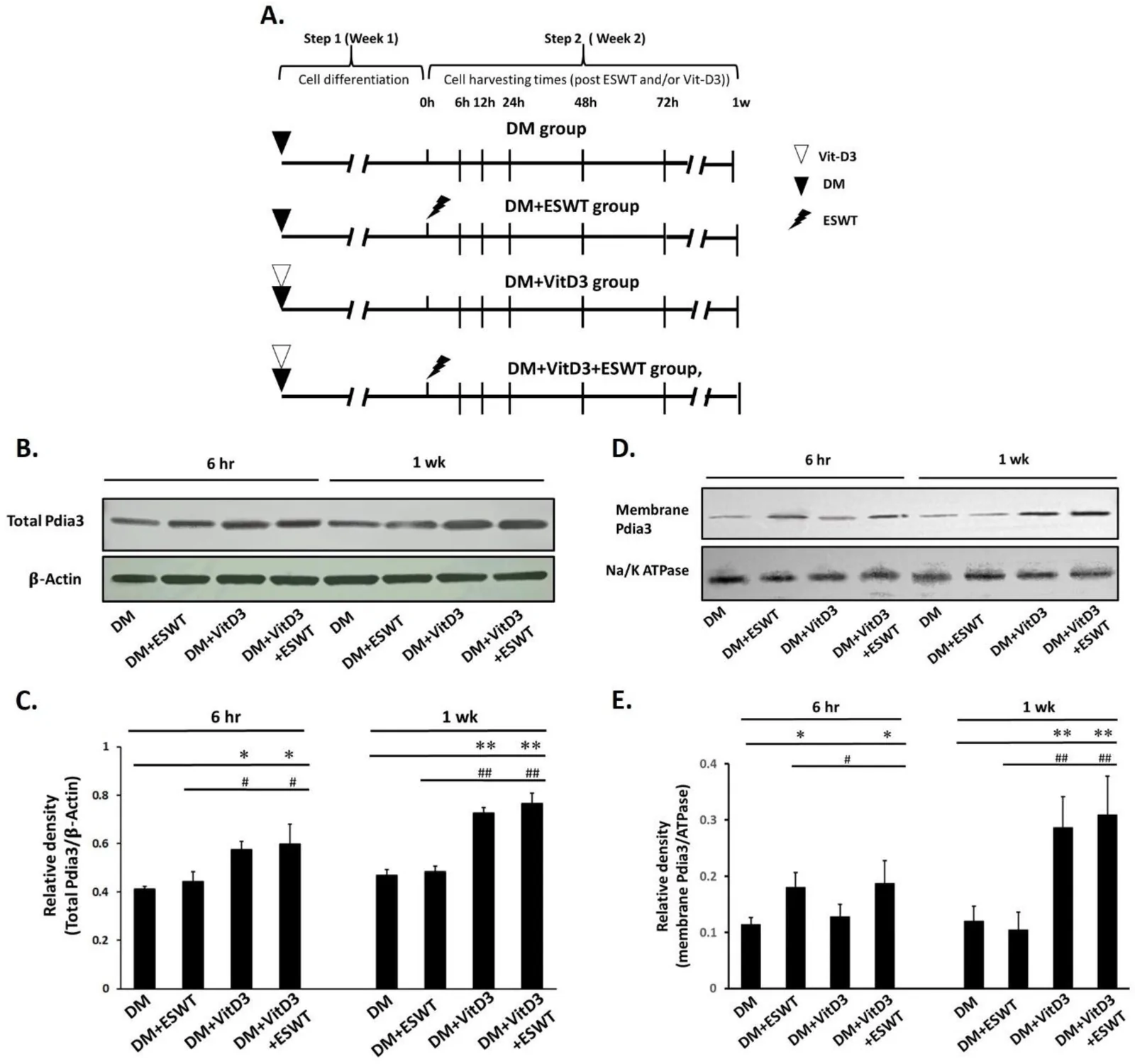
** Effects of ESWT and vitamin D3 on total cellular Pdia3 expression and membrane-localized Pdia3 in MC3T3-E1 cells.** (A) Schematic diagram of the experimental design. MC3T3-E1 cells were cultured in differentiation medium (DM) or DM supplemented with VitD3 for the first week (cell differentiation, step 1). In the second week, the cells were subjected to ESWT or no treatment, followed by cell harvesting at specific time points (cell harvesting, step 2). The four experimental groups were designated DM, DM+ESWT, DM+VitD3, and DM+VitD3+ESWT. (B) Western blot analysis and (C) quantification of total Pdia3 expression in the DM, DM+ESWT, DM+VitD3, and DM+VitD3+ESWT groups at 6 hours and 1 week after ESWT treatment. (D) Western blot analysis and (E) quantification of membrane Pdia3 expression in the same groups at the same time points. The data are presented as the means ± standard deviations (n=3). The statistically significant were at ^*^P < 0.05 and ^**^P < 0.01.

We examined whether Pdia3 expression and distribution in osteoblasts cultured with DM were influenced by ESWT, VitD3, or their combination (Figure 2). Whole cell lysates and plasma membrane extracts were analyzed at 6 hr and 1 wk after treatment. Compared with the DM group, the DM+VitD3 (P < 0.05 and P < 0.01) and DM+VitD3+ESWT (P < 0.05 and P < 0.01) groups presented significantly increased Pdia3 expression at both time points, whereas the DM+ESWT group presented no significant change (Figure 2B and 2C).

In the plasma membrane, the DM+ESWT (P < 0.05) and DM+VitD3+ESWT (P < 0.05) groups presented significantly increased Pdia3 levels at 6 hr, whereas the DM+VitD3 group did not (Figure 2D and 2E). At 1 wk, the DM+VitD3 (P < 0.01) and DM+VitD3+ESWT (P < 0.01) groups presented significantly greater plasma membrane Pdia3 levels than the DM and DM+ESWT groups. These results suggest that VitD3 primarily increased total cellular Pdia3 expression at both 6 hr and 1 wk, while ESWT mainly enhanced the localization of Pdia3 to the plasma membrane during the early phase. The combined treatment increased both total Pdia3 expression and membrane-associated Pdia3 levels, supporting the concept that ESWT facilitates the membrane translocation of VitD3-induced Pdia3 during osteoblast differentiation (Figure 2C and 2E). The combined treatments enhanced Pdia3 expression and its localization to the cell membrane, supporting the notion that ESWT facilitates the VitD3-induced membrane translocation of Pdia3, thereby promoting osteoblast differentiation.

### Effects of ESWT on the membrane distribution of Pdia3 at different time points during osteoblast differentiation

**Figure 3:**
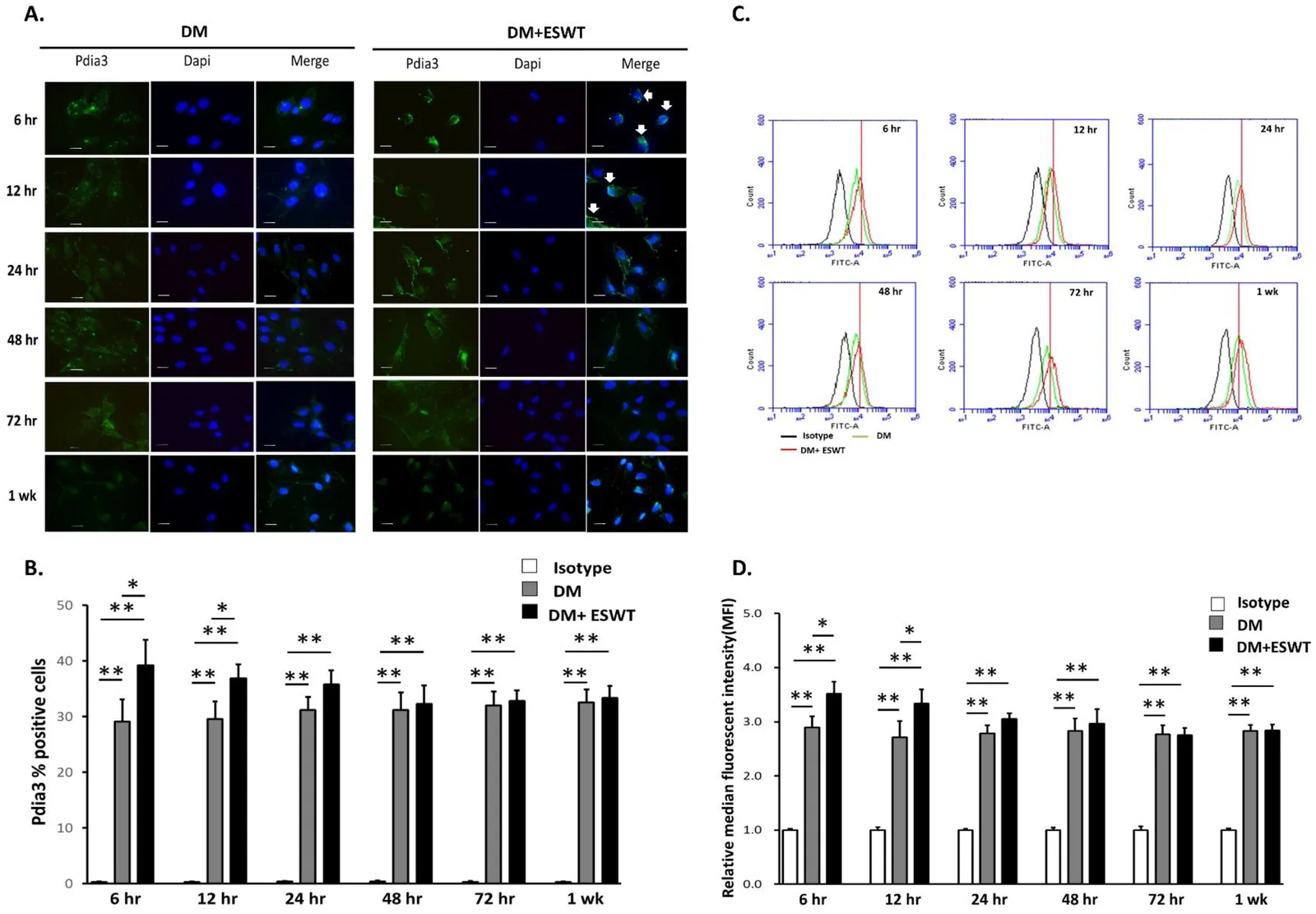
** Membrane Pdia3 localization and expression in the DM and DM+ESWT groups.** (A) Immunofluorescence analysis showing the effects of membrane Pdia3 (green) translocation (white arrow) in MC3T3-E1 cells treated with DM and DM+ESWT for 6 hours or 1 week. Nuclei were stained with DAPI (blue). The scale bar is 20 μm. (B) Bar graph depicting the percentage of Pdia3-positive cells among the isotype, DM, and DM+ESWT groups. The data are presented as the means ± standard deviations (n=3). (C) Flow cytometry histograms overlaying Pdia3 expression levels in MC3T3-E1 cells from the isotype control, DM, and DM+ESWT groups. (D) Bar graph comparing the relative mean fluorescence intensity (MFI) of Pdia3 in the isotype, DM, and DM+ESWT groups. DM refers to differentiation medium. The statistically significant were at ^*^P < 0.05 and ^**^P < 0.01.

In addition, we further investigated that membrane Pdia3 was increasing in osteoblasts after ESWT by using plasma membrane protein-specific immunofluorescence staining (Figure 3A) and flow cytometry (Figure 3C). The results showed that Pdia3 expression in the plasma membrane of osteoblasts was significantly increased in the DM+ESWT (P < 0.05) group by compared to the DM group at 6 and 12 hr (Figure 3A and 3B). In addition, through the MFI levels of Pdia3 by flow cytometry assay (Figure 3C and 3D), we found that DM+ESWT (P < 0.05) group were significantly higher than DM group at 6 hr and 12 hr. These results demonstrated that ESWT could promote the shuttling of Pdia3 from the cytoplasm to the plasma membrane in osteoblasts with early phase stimulation.

### Profile of membrane Pdia3 modulation by ESWT and VitD3 in osteoblast differentiation

**Figure 4:**
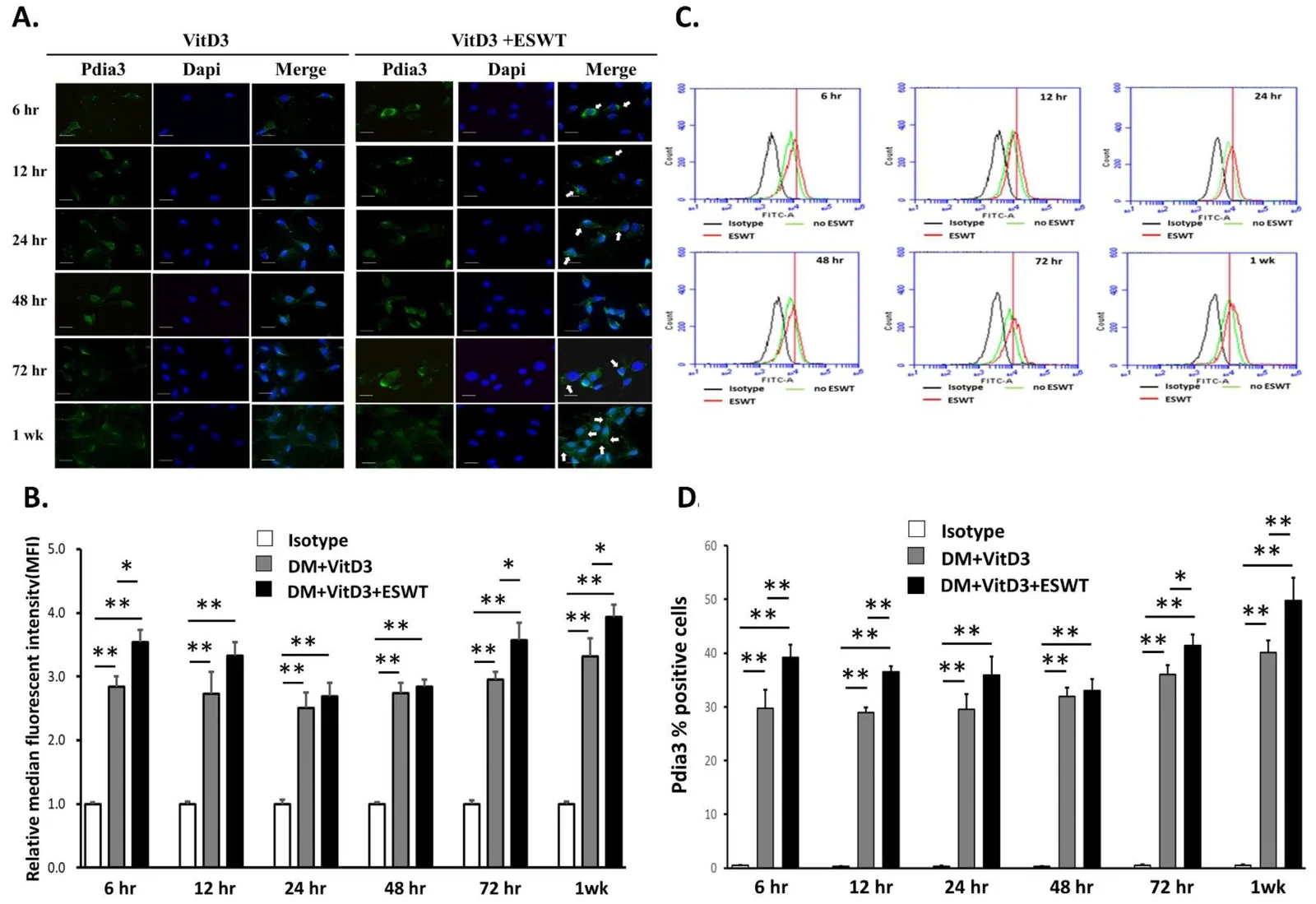
** Membrane Pdia3 translocation and expression in the DM+VitD3 and DM+VitD3+ESWT groups.** (A) Immunofluorescence analysis demonstrating membrane Pdia3 (green) translocation (indicated by white arrows) in MC3T3-E1 cells in the DM+VitD3 and DM+VitD3+ESWT groups at 6 hours and 1 week. Nuclei were stained with DAPI (blue). The scale bar is 20 μm. (B) Flow cytometry histograms overlaying Pdia3 expression levels in MC3T3-E1 cells from the isotype control, DM+VitD3, and DM+VitD3+ESWT groups. (C) Bar graph showing the relative mean fluorescence intensity (MFI) of Pdia3 compared among the isotype control, DM+VitD3, and DM+VitD3+ESWT groups. (D) Bar graph comparing the percentage of Pdia3-positive cells among the isotype control, DM+VitD3, and DM+VitD3+ESWT groups. The data are presented as the means ± standard deviations (n=3). The statistically significant were at ^*^P < 0.05 and ^**^P < 0.01.

The distributions of Pdia3 during osteoblast differentiation were measured after VitD3 treatment and combination with ESWT. In the plasma membrane protein-specific immunofluorescence staining (Figure 4A) and quantitative analysis of the positive cell (Figure 4B), Pdia3 was observed on the plasma membrane of osteoblasts in the DM+VitD3+ESWT (P < 0.05 and P < 0.01) group at 6 hr, 12 hr, 72 hr and 1 wk compared with the DM group. In addition, the DM+VitD3+ESWT (P < 0.05 and 0.01) group presented significantly more membrane-bound Pdia3 in osteoblasts at 6 hr, 12 hr, 72 hr and 1 wk compared with the DM group (Figure 4C and 4D, MFI) by plasma membrane protein-specific flow cytometry. These results indicated that VitD3 could co-activation with ESWT to enhance membrane-bound Pdia3 in the osteoblast differentiation during the early and late phase periods.

### The combined effect of ESWT and VitD3 in activating the Pdia3-PKC signaling pathway

**Figure 5:**
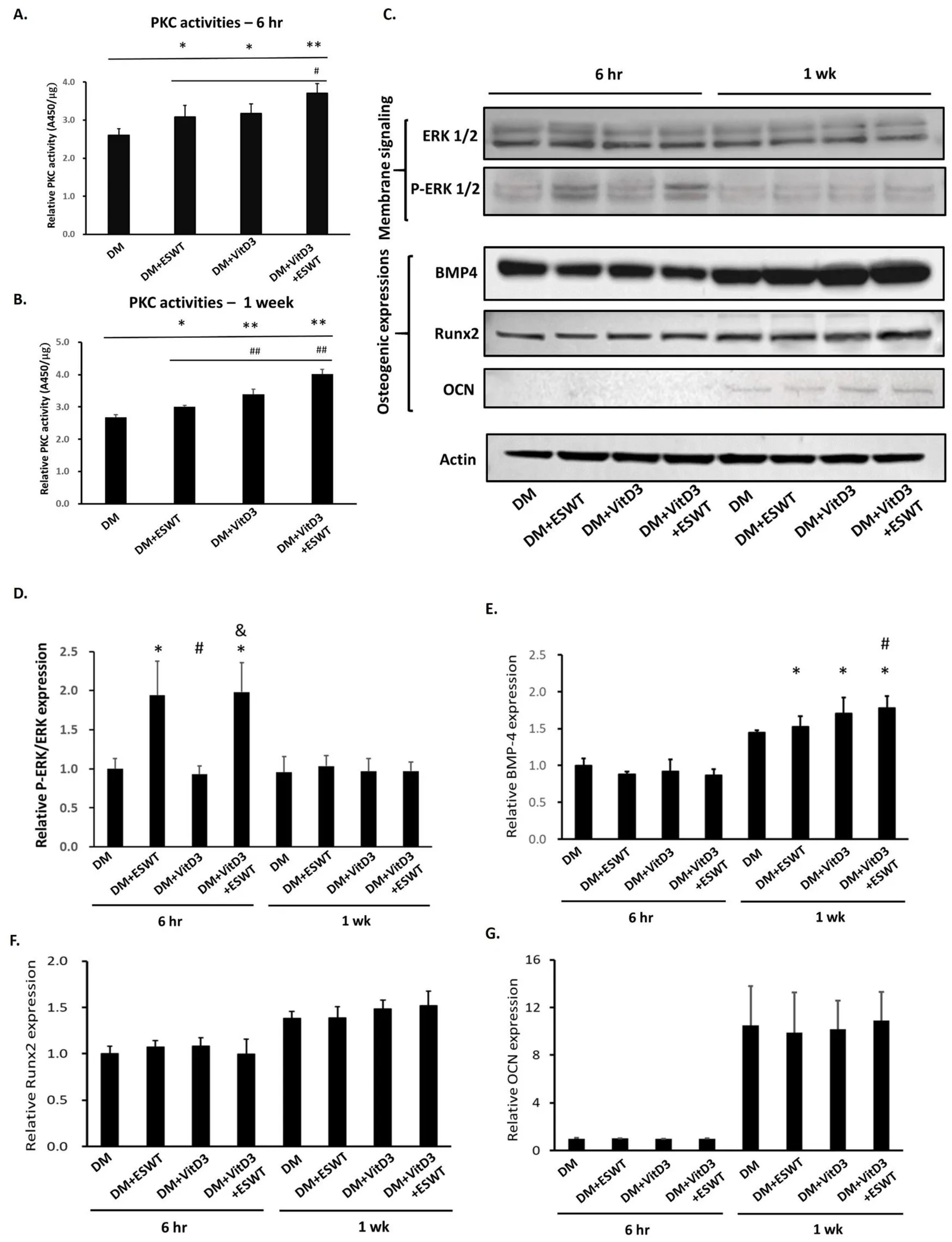
** The Pdia3-PKC signaling factors and osteogenic factors in the DM, DM+ESWT, DM+VitD3 and DM+VitD3+ESWT groups.** (A) The relative PKC activities of each group were detected at 6 hours (hr). (B) The relative PKC activities of each group were detected at 1 week (wk). (C) The expression of total ERK1/2 and p-ERK1/2 as well as the osteogenic factors BMP4, Runx2, and OCN at 6 hr and 1 wk. β-actin was used as an internal control. The relative expression of (D) p-ERK1/2 over ERK 1/2, (E) BMP4, (F) Runx2 and (G) OCN at 6 hr and 1 wk in each group. The data are presented as the means ± standard deviations (n=3). An actin protein is the internal control. Statistical significance was set at ^*^P < 0.05 compared with the DM group. ^#^P < 0.05 for DM+VitD3 and DM+VitD3+ESWT compared with DM+ESWT. ^&^P < 0.05 compared between DM+VitD3 and DM+VitD3+ESWT.

The Pdia3-PKC signaling pathway is activated by VitD3 to regulate bone formation. Here, the results were demonstrated that ESWT could activate the Pdia3-PKC signaling pathway but also had combined effects with VitD3 to promote the maturation of osteoblasts in early (6 hr) and late phase periods (1 wk). Compared with the DM group, the DM+ESWT, DM+VitD3 and DM+Vit3D+ESWT groups presented significantly increased PKC activity at 6 hr (P < 0.05, P < 0.05 and P < 0.01) and 1 wk (P < 0.05, P < 0.01 and P < 0.01) (Figure 5A and 5B). The combined effect of the DM+VitD3+ESWT group at 6 hr (P < 0.05) and 1 wk (P < 0.01) on PKC activity was significantly greater than that of the DM+ESWT and DM+VitD3 groups. Phospho-ERK1/2 signaling was detected at 6 hr, and that in the DM+ESWT (P < 0.05) and DM+Vit3D+ESWT (P < 0.05) groups were higher than that in the DM+VitD3 and DM groups (Figure 5C and 5D). The expression levels of the osteogenic protein BMP4 significantly increased at 1 wk (P < 0.05) (Figure 5E, DM+ESWT, DM+Vit3D and DM+VitD3+ESWT groups), and Runx2 and OCN did not show difference at these time points (Figure 5F and 5G). These results indicated that VitD3 could act together with ESWT to activate the Pdia3-PKC signaling pathway earlier and promote BMP4 expression in the late phase period for osteoblast maturation.

### F-actin plays a significant role in increasing membrane Pdia3 levels via ESWT-stimulated repolymerization

Previous studies have suggested that Pdia3 interacts with F-actin during calcium uptake. In the experiment, F-actin appeared as broken filaments (depolymerization) at 6 to 12 hr after ESWT, but its rod-like formation (repolymerization) was restored after 24 hr (Supplemental Figure 2). Correspondingly, Pdia3 exhibited aggregated, thickened formations at the cell peripheries between 6 to 24 hr, which subsided and merged into uniformly distributed membrane proteins by 48 hr. These observations suggest that Pdia3 interacts with F-actin and moves to the plasma membrane.

**Figure 6:**
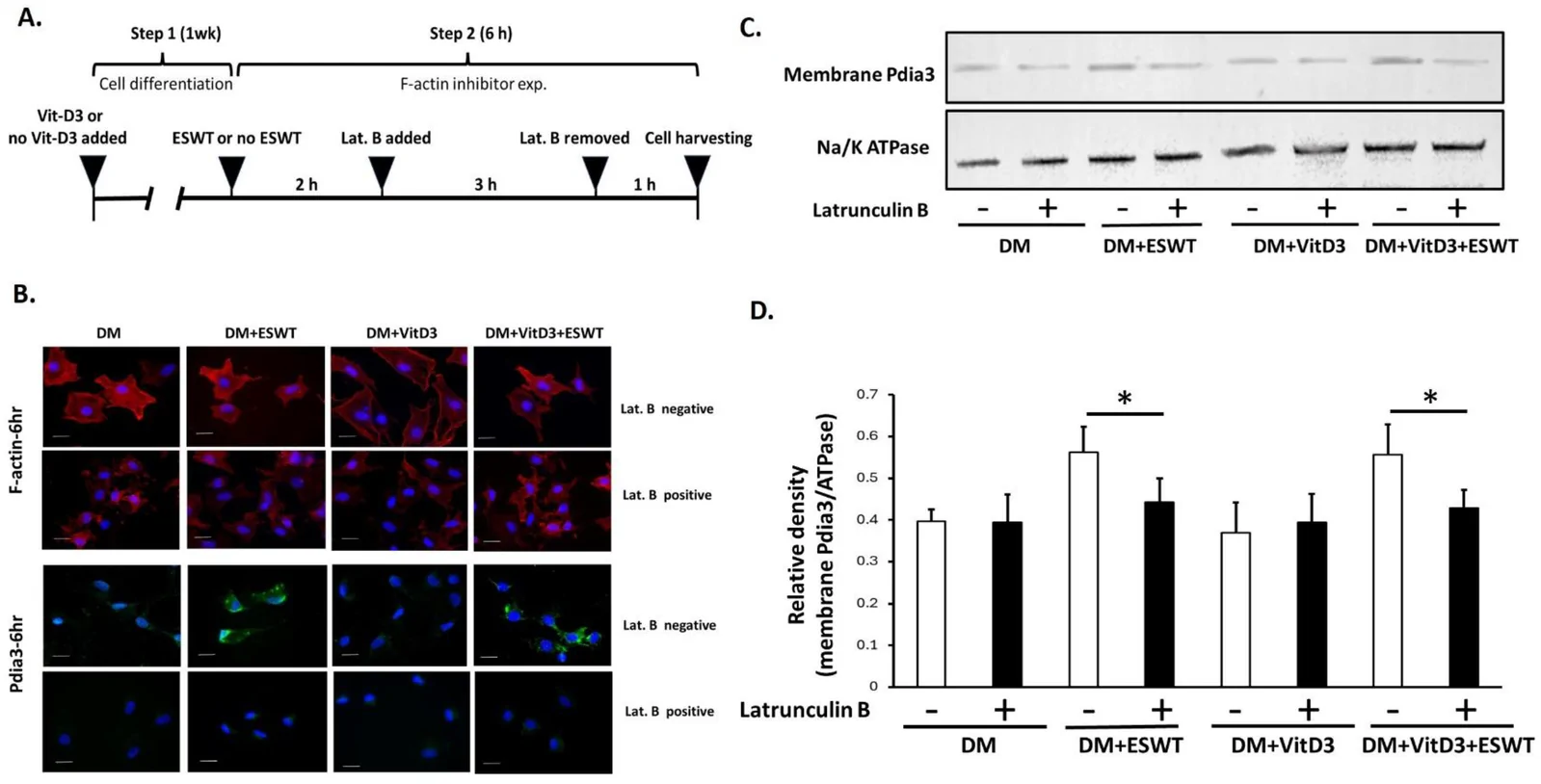
** Effect of F-actin polymerization inhibition on membrane Pdia3 translocation.** (A) Timeline for the addition and removal of latrunculin B (Lat. B) in the cell culture experiment. (B) Effects of F-actin and the Pdia3 membrane in the presence and absence of Lat. B in all groups at 6 hours in each group. The scale bar is 20 μm. (C) Western blot and (D) quantification results comparing Lat. B positive and negative. A Na^+^/K^+^-ATPase is the internal control for membrane protein and the expression is not changed after treatments. The data are presented as the means ± standard deviations (n=3). The statistical significance is at ^*^P < 0.05.

According the experiment design (Figure 6A), the fluorescence microscopy (Figure 6B) and western blot assays (Figure 6C and 6D) demonstrated that ESWT stimulated the redistribution of Pdia3 within the plasma membrane in the DM+ESWT and DM+VitD3+ESWT groups. In addition, latrunculin B (an actin polymerization inhibitor) inhibited F-actin polymerization after ESWT, resulting in blocked redistribution of Pdia3 to the cell membrane in the DM+ESWT (P < 0.05) and DM+VitD3+ESWT (P < 0.05) groups (Figure 6B, 6C and 6D; latrunculin B treatment). These results demonstrated that ESWT promoted the transport of Pdia3 to the plasma membrane by stimulating F-actin repolymerization.

## Discussion

The novelty of the current study lies in identifying Pdia3 as a potential mediator linking VitD3 signaling and ESWT-induced mechanotransduction in osteoblasts. Although Pdia3 is known to participate in rapid vitamin D signaling, its involvement in ESWT-triggered osteogenic responses has not previously been clarified. We demonstrate that ESWT promotes the translocation of Pdia3 to the plasma membrane through F-actin repolymerization, revealing a previously unrecognized mechanism by which mechanical stimulation regulates membrane receptor localization. Importantly, our results indicate that ESWT and VitD3 exert distinct effects on Pdia3 functional regulation. VitD3 predominantly increased total cellular Pdia3 expression, whereas ESWT primarily promoted the redistribution of cytosolic Pdia3 to the plasma membrane through F-actin-dependent trafficking. The combined treatment enhanced the membrane-associated localization of Pdia3, thereby activating downstream PKC signaling and promoting osteoblast differentiation and mineralization. By integrating mechanotransduction and vitamin D signaling pathways, this study provides novel mechanistic insight into how biochemical and mechanical stimuli converge at the level of membrane signaling and cytoskeletal remodeling.

VitD3 is known to regulate bone metabolism, and Pdia3 is one of the receptors that initiates the rapid membrane-associated Pdia3-PKC signaling pathway by VitD3 10. Pdia3 present in caveolae, where it interacts with PLA2-activating protein (PLAA) and caveolin-1 to initiate rapid signaling by PLA2, PLC, PKC, and ultimately the ERK1/2 family of mitogen-activated protein kinases (MAPKs) 10, 33. In this study, VitD3 treatment significantly increased Pdia3 expression and plasma membrane localization at both 6 hr and 1 wk. The combination of VitD3 and ESWT increased Pdia3 expression, PKC activity, and phospho-ERK1/2 levels, demonstrating that these treatments could cooperatively activate the Pdia3-PKC signaling axis (Figure 5). Disruption of the PDIA3 protein attenuates PKA and PKC signaling and calcium influx 12. Furthermore, the combined effects of VitD3 and ESWT were evident in the upregulation of BMP4 expression, which participated to early osteogenic signaling (Figure 5E). VitD3 combined with ESWT could be provided a mechanistic basis for future studies in bone regeneration.

Interestingly, although BMP4 expression was significantly increased following VitD3 and ESWT treatment, Runx2 and OCN expression did not significantly differ at the examined time points. This finding may reflect the temporal heterogeneity of osteogenic marker expression during osteoblast differentiation. Runx2 is primarily involved in the early commitment phase of osteogenesis and may not remain elevated during later stages, whereas OCN is generally regarded as a late marker associated with matrix maturation and mineralization 34, 35. Because Runx2 and OCN were evaluated only at 6 hr and 1 wk after treatment, their higher expression may not have been captured within the selected observation window. The marked increases in ALP activity, BMP4 expression, PKC activation, and mineral deposition nevertheless support the overall osteogenic effects of VitD3 and ESWT. Future studies incorporating additional intermediate and late time points may further clarify the temporal regulation of osteogenic markers in response to combined VitD3 and ESWT treatment.

Osteoblasts are mechanosensitive cells that promote new bone formation and maintain bone homeostasis. Various mechanical forces can promote the proliferation and differentiation of osteoblasts 36. Numerous *in vitro* studies have also shown that mechanical forces can significantly alter the cytoskeleton, morphology, and volume of osteoblasts, including changes in cell and nuclear structure 37. ESWT is a mechanical, acoustic shock wave that induces multiple forces and has emerged as a potent mechanotransductive stimulus for the regeneration of many tissues, especially for bone formation 22, 38. Several studies have reported that ESWT induces osteogenic differentiation through the activation of mechanotransduction signaling pathways in mesenchymal stem cells and osteoblasts 39, 40. The current results showed that ESWT enhanced osteoblast differentiation by stimulating ALP activity and calcium deposition (Figure 1). Moreover, both ESWT alone and the combined VitD3+ESWT treatment promoted the redistribution of Pdia3 to the plasma membrane as early as 6 hr, with effects persisting through 24 hr (Figure 3). ESWT and VitD3 combined with ESWT-induced Pdia3 redistribution was dependent on F-actin polymerization, further underscoring the mechanotransductive role of cytoskeletal remodeling in osteoblast differentiation.

The present findings emphasize the crucial role of mechanical forces in modulating osteoblast differentiation through structural remodeling of cellular components. ESWT-induced mechanical stimulation was shown to regulate the polymerization and depolymerization of F-actin, an important cytoskeletal component, thereby facilitating the redistribution of Pdia3 to the plasma membrane (Figures 2 and 3). This dynamic interaction between F-actin and Pdia3 highlights the importance of actin cytoskeletal remodeling in mechanotransduction processes. Additionally, the use of latrunculin B, an actin polymerization inhibitor, effectively blocked the ESWT-induced redistribution of Pdia3, further validating the role of F-actin (Figure 6C). Notably, caveolae-1, a membrane-associated protein known for its role in mechanosensing and signal transduction, may also play a part in this context. Previous studies have demonstrated that caveolae-1 interacts with Pdia3 and actin filaments to regulate the localization of signaling proteins during mechanical stimulation 14, 41. Although this study did not directly investigate this phenomenon, caveolae-1 may plausibly contribute to the increased membrane-bound Pdia3 levels observed after ESWT by stabilizing membrane domains or facilitating protein trafficking. Further research is needed to confirm and elucidate this potential mechanism (Graphical abstract).

### Study limitations

Although the present study demonstrated the combined effects of active VitD3 and ESWT on osteoblast differentiation and mineralization, several limitations should be acknowledged. The use of MC3T3-E1 cells may not fully replicate the complexity of *in vivo* bone environments; future studies should include animal models or clinical trials to validate these findings. The assessment of only short-term outcomes limits the interpretation of the long-term effects on bone regeneration. Additionally, while the Pdia3-PKC signaling pathway was identified, other pathways potentially critical to osteoblast differentiation were not explored. The reliance on specific ESWT parameters and VitD3 concentrations may restrict the generalizability of these findings across different treatment protocols, experimental settings, and patient populations. Moreover, while the combined treatment consistently resulted in higher osteogenic responses than either intervention alone, these findings should not be interpreted as definitive evidence of a synergistic or universally superior effect. Further studies are required to validate these observations under diverse experimental conditions and to establish their translational relevance. A rigorous determination of synergy requires quantitative modeling approaches, such as Bliss independence or Loewe additivity analyses, which were not performed in the present study. Therefore, it remains unclear whether the combined effects exceed the expected additive effects of the individual treatments. Future studies incorporating dose-response designs and formal synergy modeling are warranted to clarify the nature of the interaction between VitD3 and ESWT. Furthermore, while the role of F-actin in Pdia3 dynamics has been studied, other cytoskeletal components have not been investigated.

## Conclusion

This study showing the novel combined effects of ESWT and VitD3 in enhancing osteoblast differentiation and mineralization, revealing critical molecular mechanisms underlying bone formation. The combination of ESWT and VitD3 significantly increased ALP activity, calcium deposition, and the expression of BMP4, demonstrating their combined potential to promote osteoblast maturation. Importantly, this research identified that Pdia3 may contribute to osteoblast differentiation, as its expression and membrane localization are increased when it is stimulated by ESWT and VitD3. Additionally, the interaction between Pdia3 and F-actin, which is mediated by ESWT-induced cytoskeletal remodeling, underscores the mechanistic basis of Pdia3 shuttling to the plasma membrane. These findings demonstrate the potential of combined VitD3 and ESWT treatment to promote bone regeneration, offering new insights into osteoblast biology and mechanism of the bone regeneration.

## Supplementary Material

Supplementary figures.

## Abbreviations

VitD₃: 1α,25(OH)₂D₃
ESWT: Extracorporeal shockwave therapy
PDI: protein disulfide isomerase
ER: endoplasmic reticulum
ILF3: interleukin enhancer-binding factor 3
DKC1: dyskerin pseudouridine synthase 1
PLC: phospholipase C
PKC: protein kinase C
ERK1/2: extracellular signal-regulated kinases 1 and 2
VDR: vitamin D receptor
OPN: osteopontin
ALP: alkaline phosphatase
VEGF: vascular endothelial growth factor
PCNA: proliferating cell nuclear antigen
eNOS: endothelial nitric oxide synthase
BMP2: bone morphogenetic protein 2
FAK: Focal adhesion kinase
TGF-β1: Transforming growth factor beta-1
IGF-1: insulin-like growth factor 1
Wnt5a: Wnt Family Member 5a
DM: differentiated medium
ALP: alkaline phosphatase
GM: growth medium
FACS: fluorescence-activated cell sorting
MTT: 3-(4,5-Dimethylthiazol-2-yl)-2,5-diphenyltetrazolium bromide
MFIs: mean fluorescent intensities
PLAA: PLA2-activating protein
MAPKs: mitogen-activated protein kinases
