# Supplementary material for: Combined Effects of Vitamin D3 and Extracorporeal Shockwave Therapy on Osteoblast Differentiation via F-actin mediated Pdia3 Membrane Localization
Source: Int J Med Sci. 2026 Jul 22;23(9):2767–79. doi: 10.7150/ijms.127831 (PMC13539911; doi:10.7150/ijms.127831)
Supplement: Supplementary file 1 [file ijmsv23p2767s1.pdf]

Supplemental Figure Legends

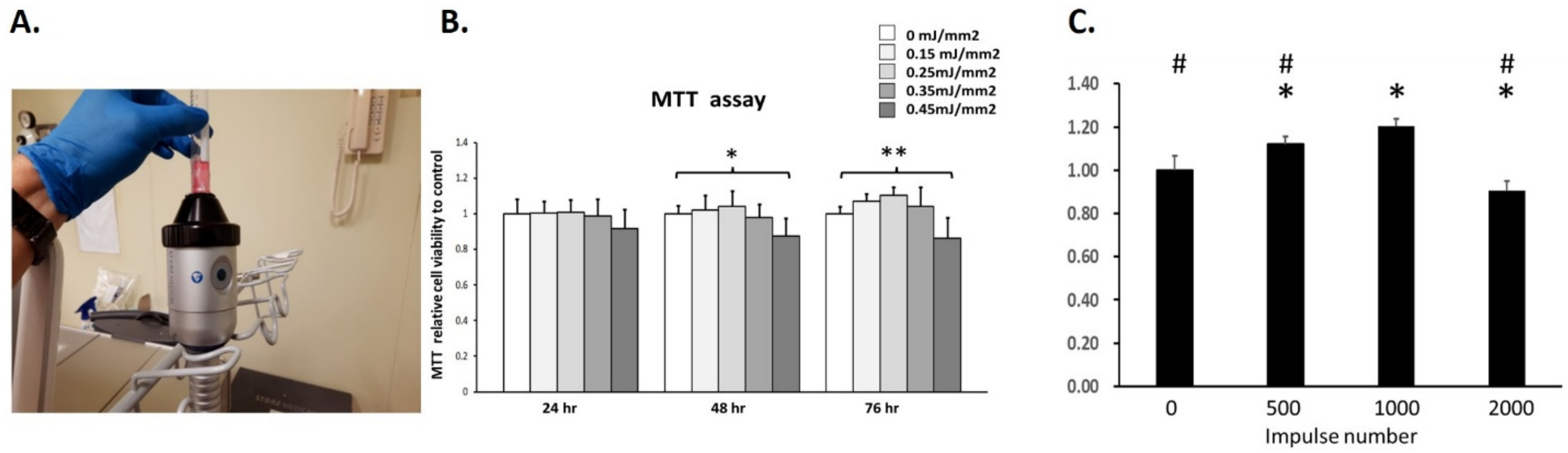

Supplemental Figure 1. The optimal dosage of ESWT for osteoblast differentiation was determined. (A) A photograph showing the ESWT applied to the cells with medium by a 3 M membrane with air sealed in a 15 mL tube. (B) MTT assay at 24, 48 and 72 hours under various conditions by ESWT. Statistical significance is denoted by $^{*}P < 0.05$ and $^{**}P < 0.01$ compared with 0 mJ/mm^2, 1000 impulses, and 4 Hz. (C) Dose-response analysis showing the influence of different doses 0.25 mJ/mm^2 with 0, 500, 1000, and 2000 impulses, 4 Hz. The results are statistically significant when $^{*}P < 0.05$ compared with 0 impulses, 4 Hz and $^{\#}P < 0.05$ compared with 1000 impulses, 4 Hz. Each group is repeated at least three times.

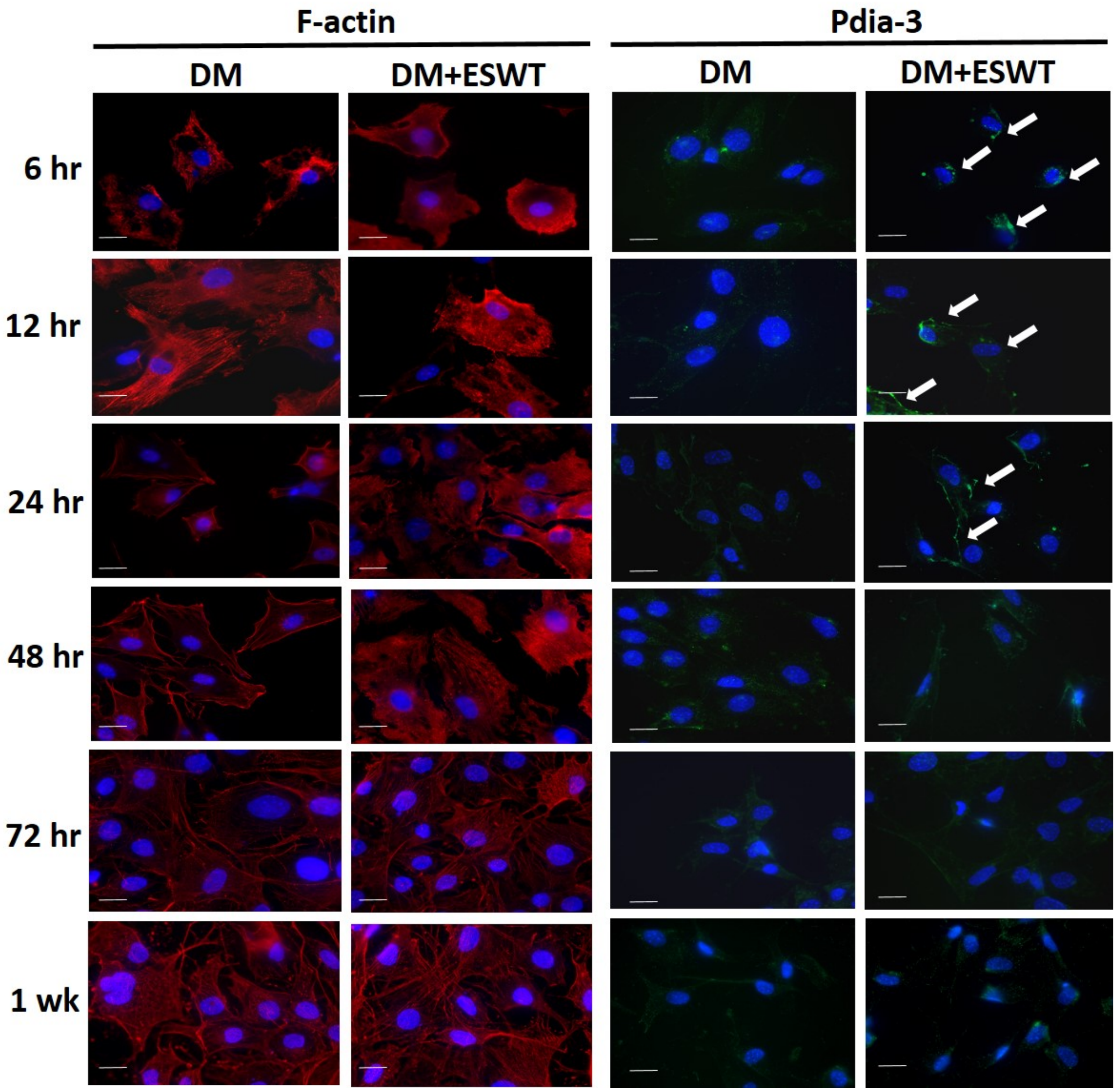

Supplemental Figure 2. The presence of membrane Pdia3 in the DM and ESWT groups is studied at 6 hours to 1 week after ESWT. Effects of F-actin (red) depolymerization and repolymerization and membrane Pida3 (green, white arrows) translocation, as shown by immunofluorescence staining. The blue color represents the cell nucleus as determined by DAPI staining. The scale bar is 20 μm.
